# Closing the Evidence Gap of Cash Transfer for Tuberculosis-Affected Households: Comment on "Does Direct Benefit Transfer Improve Outcomes Among People With Tuberculosis? – A Mixed-Methods Study on the Need for a Review of the Cash Transfer Policy in India"

**DOI:** 10.34172/ijhpm.2022.7658

**Published:** 2022-11-23

**Authors:** Ahmad Fuady

**Affiliations:** ^1^Department of Community Medicine, Faculty of Medicine Universitas Indonesia, Jakarta, Indonesia.; ^2^Primary Health Care Research and Innovation Center, Indonesian Medical Education and Research Institute, Faculty of Medicine Universitas Indonesia, Jakarta, Indonesia.

**Keywords:** Cash Transfer, Financial Protection, Nutrition, Socioeconomic Support, Tuberculosis

## Abstract

Achieving the targets of eliminating tuberculosis (TB) requires a combination of biomedical, epidemiological, and social approaches. Having hitted by the coronavirus disease 2019 (COVID-19) pandemic which diminishes the financial capacity of TB-affected households, the importance of delivering socioeconomic support to TB-affected household emerges. However, the evidence of TB-related socioeconomic support is still scarce, and some questions are left unanswered. A sequential explanatory mixed-methods study by Dave and Rupani shows that the direct benefit transfer (DBT), a form of cash transfer, to TB-affected households improves TB treatment outcomes in India despite the challenges. Some critical issues remain to be discussed: trading-off between the amount of cash and its sustainability, choosing the most appropriate support packages, detecting, and reaching the target population, and arranging the most effective delivery strategy. Knowledge gap remains to be answered, and a global research agenda and political commitment are critical to encourage more evidence in delivering socioeconomic support for TB control.

 Developing strong socioeconomic support for households affected by tuberculosis (TB) is inevitable to accelerate TB elimination. Having slow progress of declining global TB incidence rate in the last decade,^1^ also hampered by the coronavirus disease 2019 (COVID-19) pandemic,^2^ the global efforts to achieve the end TB goals are out of track. The COVID-19 pandemic, in particular, also diminishes the financial capacity of TB-affected households. The incidence of TB-related catastrophic costs in low- and middle-income countries (LMICs) and TB high burden countries remains high.^3-5^ This economic burden can prolong diagnostic delays, increase the undetected and untreated TB cases, allow more TB transmission, cases, and mortality, and increase the risk of facing TB-related catastrophic costs.^6^

 Therefore, the efforts to eliminate TB should be refocused using new tools and knowledge and expanding to socioeconomic interventions,^7^ of which are critical in achieving the World Health Organization’s (WHO’s) End TB Strategy targets to eliminate the proportion of TB-affected households facing catastrophic costs. Preventing such catastrophic costs by providing socioeconomic support is also vital to achieving other targets: reducing TB incidence and mortality, since facing catastrophic costs is closely related to poor TB outcomes.^8,9^

 Despite the increasing awareness and commitment to delivering socioeconomic support for TB-affected households, evidence of TB-related socioeconomic support is still scarce. Brazil, with its Bolsa Familia Program, has the most evidence despite the less rigorous evaluation methods. Some trials, primarily conditional cash transfers, have tried to close the evidence gap.^10-13^ However, many questions are still left unanswered: target population (TB-specific, inclusive, or sensitive population), conditionality (conditional/unconditional), forms (cash transfer, nutritional package, travel voucher), strategy (hard cash, voucher, bank transfer), amount of support, and sustainability.

 A sequential explanatory mixed-methods study by Dave and Rupani contributes an evidence that the direct benefit transfer (DBT) to TB-affected households improves TB treatment outcomes in India.^14^ This program is a TB-specific initiative which has targeted TB-related households as the beneficiaries to tackle the dual epidemic of under-nutrition and TB in India. The INR 500 (~US$ 7) per month cash transfer for nutritional support during the six-month TB treatment showed a significant effect: the program’s beneficiaries were less likely to have unfavourable treatment outcomes—defined as lost-to-follow-up, failure, and death.

 Besides evaluating the outcomes quantitatively, this study provides insightful qualitative evidence on the challenges of delivering cash transfers in a high TB burden and LMIC. A TB-specific cash transfer, compared to TB-sensitive and inclusive initiatives,^15^ may be simpler and easier to manage. However, some critical issues remain to be discussed, particularly for replication and scaling up to the broader setting.

## Little Money, High Impact?

 The challenge of cash transfer is trading-off between the amount of support and its sustainability. The study noted that almost all the patients perceived insufficient monetary benefit to meet their nutrition needs. At the same time, other studies showed that nutritional supplements contributed to a high proportion of household spending that could be catastrophic.^1,3,16^ The more severe people feel the disease, the more they perceive that they need nutritional supplementation.

 Besides addressing the need for nutrition support, it is also critical to address income loss related to TB. The incidences of TB-related catastrophic costs are high, and income loss significantly contributes to the total costs. Moreover, with a wide range of households’ financial capacities, medical and nutrition needs, and income loss, it is difficult to determine the amount of a cash transfer that would fit all patients.

 The money provided by cash transfer is little. There are various cash support schemes that the government has previously delivered, such as National Social Assistance Program as financial and food assistance to the elderly, widows, and persons with disabilities.^17^ This program provides several cash support schemes varying from INR 200 to 500 (US$ 2.5 to 7) per month per beneficiary and/or 10 kg of food grains per month. These are little cash. Some studies showed positive impacts of the program,^18^ but some others identified the limitations.^19,20^ The little cash could help households to survive or alleviate the economic burden. However, it is still questioned whether the little cash could solve the actual economic damage caused by chronic diseases. Instead, it is only a temporary solution, given that people with TB may face more substantial problems, such as job loss and the inability to gain a similar income level after TB diagnosis.

 While the money provided is little, increasing the monetary benefit would demand an additional government budget. For example, it can range between 46%-148%, compared to the TB control program without cash transfer.^21^ Its affordability and sustainability, therefore, should be carefully considered. One of the challenges of small-scale trials providing socioeconomic support is the scale-up to a broader scale.

 Once a small-scale cash transfer program succeeds, and its scaling-up is planned, identifying financial resources which can co-finance the support and sustain it over a long period is a must. It is time to seek multiple donors from domestic and international organizations. Despite its success on a small scale, the cash transfer could also make broader social changes beyond the initial outcome of the original small-scale cash transfer target.^22^ An (extended) cost-effectiveness analysis will be helpful to inform how cost-effective the investment is, not only in improving treatment completion but also in avoiding catastrophic costs and impoverishment.^23^

## Technical Hassle

 Despite its more straightforward mechanism, a TB-specific cash transfer, particularly in LMICs, could still be hampered by limited resources. For example, the DBT program distributes cash via bank account, that can help to avoid moral hazards and reduce the risk of stigmatization. However, households in LMICs, particularly low-income households, often have problems with their bank account and complex beneficiary verification. Their bank account may be inactive, or even they have no bank account and required documents to open the account. This problem can complicate the delivery of cash transfers, including a potentially delayed payment and the program evaluation.

 Delivering the actual cash may also be problematic. It leaves the possibility of fraud and misappropriation of cash. Logistically, for healthcare workers or technical officers, it will also be easier to give the actual cash in one batch, mainly at the end of the month. However, some people need the cash and cannot wait until the end of the month, which could lower their adherence to the treatment and support.

 The challenge in a limited resources setting is the error in detecting beneficiaries because of poor administrative records. It is almost impossible to reach all potential beneficiaries, while people who are not recorded in civil administration offices or lack official documents are those who require the support the most. They include very the poor, homeless, unemployed people, and migrants. A combination of socioeconomic support or alternative support for those unreachable, for example, a nutrition package or food kit for the homeless, would be helpful to reach them without delay.

 There have still been knowledge gaps in answering the remaining challenges. A global research agenda and political commitment are required to encourage more production of evidence in delivering socioeconomic support.

## Ethical issues

 Not applicable.

## Competing interests

 Author declares that he has no competing interests.

## Author’s contribution

 AF is the single author of the paper.
